# Kallikrein-related peptidase 8 is expressed in myocardium and induces cardiac hypertrophy

**DOI:** 10.1038/srep20024

**Published:** 2016-01-29

**Authors:** Buqing Cao, Qing Yu, Wei Zhao, Zhiping Tang, Binghai Cong, Jiankui Du, Jianqiang Lu, Xiaoyan Zhu, Xin Ni

**Affiliations:** 1 Department of Physiology, Second Military Medical University, Shanghai 200433, China; 2 School of Kinesiology, The key Laboratory of Exercise and Health Sciences of Ministry of Education, Shanghai University of Sport, Shanghai 200438, China

## Abstract

The tissue kallikrein-related peptidase family (KLK) is a group of trypsin- and chymotrypsin-like serine proteases that share a similar homology to parent tissue kallikrein (KLK1). KLK1 is identified in heart and has anti-hypertrophic effects. However, whether other KLK family members play a role in regulating cardiac function remains unknown. In the present study, we demonstrated for the first time that KLK8 was expressed in myocardium. KLK8 expression was upregulated in left ventricle of cardiac hypertrophy models. Both intra-cardiac adenovirus-mediated and transgenic-mediated KLK8 overexpression led to cardiac hypertrophy *in vivo*. In primary neonatal rat cardiomyocytes, KLK8 knockdown inhibited phenylephrine (PE)-induced cardiomyocyte hypertrophy, whereas KLK8 overexpression promoted cardiomyocyte hypertrophy via a serine protease activity-dependent but kinin receptor-independent pathway. KLK8 overexpression increased epidermal growth factor (EGF) production, which was blocked by the inhibitors of serine protease. EGF receptor (EGFR) antagonist and EGFR knockdown reversed the hypertrophy induced by KLK8 overexpression. KLK8-induced cardiomyocyte hypertrophy was also significantly decreased by blocking the protease-activated receptor 1 (PAR1) or PAR2 pathway. Our data suggest that KLK8 may promote cardiomyocyte hypertrophy through EGF signaling- and PARs-dependent but a kinin receptor-independent pathway. It is implied that different KLK family members can subtly regulate cardiac function and remodeling.

The tissue kallikrein-related peptidase family (KLK) is a group of trypsin- and chymotrypsin-like serine proteases that share a similar homology to parent tissue kallikrein (KLK1)^1^. KLKs are encoded by tandemly arranged genes of multigene families, which comprise 15 genes in human and 20 genes in rat^2^. KLKs are translated as pre-proenzymes, and then cleaved upon release from the secretary pathway to become proenzymes. Once secreted, pro-KLKs are further processed by other KLKs or other proteases to become active extracellular enzymes^1^,^2^. Kininogen is a well-known substrate of KLK members. Low-molecular weight kininogen is cleaved by KLK1, leading to the release of the vasoactive peptide kinin^1^,^2^. Kinin interacts with the kinin receptors to exert a broad spectrum of biological effects such as, vasodilation^3^, non-vascular smooth muscle contraction^4^,^5^ and inflammation^6^,^7^. Besides kininogens, a variety of substrates can be degraded by KLK family serine protease activity. For instance, extracellular matrix proteins including collagen and fibronectin can be degraded by both KLK family members^1^,^2^,^8^,^9^. The protelytic process of pro-epidermal growth factor (EGF) into mature EGF is mediated by KLK1^10^. In addition, protease-activated receptors (PARs) are also indentified to be the substrates of KLK family members^10^,^11^,^12^.

KLK8, which is also known as neuropsin, is a new member of KLKs. It was first identified in the mouse hippocampus^13^. KLK8 has now been identified in a variety of tissues, with highest density in skin and brain^14^. As a member of KLKs, KLK8 may exert its function by cleaving various proteins as other KLK members. Although the substrates for KLK8 remain largely unknown, it has been shown that the effect of KLK8 on skin desquamation^15^, synaptic plasticity in brain^16^ and invasiveness of tumor cells^17^ is associated with its serine protease activity.

Among KLKs, KLK1 is the most widely studied member. In myocardium, a local KLK1-kinin system has been identified^18^,^19^. Kinins have protective effects against myocardial ischemia/reperfusion insult or hypertrophy by interacting with two specific receptors, kinin B1 receptor (B_1_R) and B2 receptor (B_2_R)^20^,^21^,^22^. Abnormality in KLK1–kinin system (KKS) is associated with cardiac hypertrophy^2^,^23^. However, whether KLK8 is expressed in myocardium and its functions in the heart have not been reported. Here, we identified the expression of KLK8 in rat myocardium and investigated the expression pattern of KLK8 in hypertrophic hearts. We further explored the effects of KLK8 on cardiomyocyte hypertrophy and underlying mechanisms both *in vitro* and *in vivo*.

## Results

### KLK8 is upregulated in hypertrophic hearts

**Figure 1:**
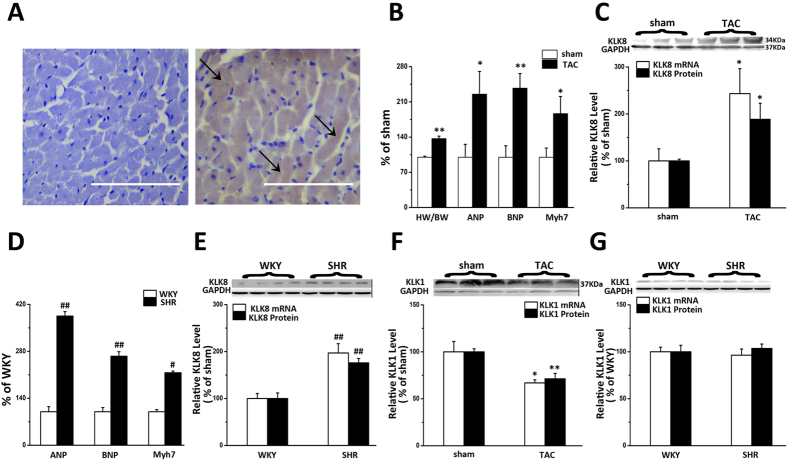
KLK8 is upregulated in hypertrophic hearts. (**A**) Expression of KLK8 in myocardium. The left panel showed negative control. Pre-absorption of the KLK8 primary antibody was performed with a 10-fold excess of the blocking peptide. The right panel is the representative immunostained section of rat left ventricular myocardium with antibody against KLK8. The positive staining was mainly localized to cardiomyocytes as indicated by arrows. Original magnification, ×400. Scale bars correspond to 100 μm. (**B,C**) showed levels of cardiac hypertrophic markers (HW/BW, ANP, BNP and Myh7) and KLK8 in myocardium of Sham and TAC rats, respectively. D and E showed levels of cardiac hypertrophic markers (ANP, BNP and Myh7) and KLK8 in myocardium of WKY and SHR rats, respectively. F and G showed KLK1 levels in myocardium of Sham/TAC and WKY/SHR rats, respectively. Data were expressed as mean percentage of Sham or WKY ± SEM (n = 7). *P < 0.05, **P < 0.01 vs Sham. ^#^*P* < 0.05, ^##^P < 0.01 vs WKY. Representative Western blotting bands are on the top of corresponding graphs.

As shown in Fig. 1A, immunoreactive KLK8 was localized in ventricular myocytes of rat heart using a primary antibody that recognizes KLK8. KLK8 Immunoreactivity was abolished when the antibody was preabsorbed with excess peptide, thereby confirming the specificity of the antibody.

We then investigated the expression pattern of KLK8 in hypertrophic hearts. Compared to the sham-operated rats, 8-week TAC rats showed significant left ventricular hypertrophy as evidenced by 1.5-fold increased heart weight to body weight (HW/BW) ratio, 2.3-fold increased ANP mRNA expression, 2.4-fold increased ANP mRNA expression, and 1.9-fold increased Myh7 mRNA expression (Fig. 1B). The mRNA and protein levels of KLK8 were significantly increased in the hearts subjected to TAC compared with Sham group (Fig. 1C, *P* < 0.05).

It is known that hypertension could result in left ventricular hypertrophy. In spontaneously hypertensive rats (SHR), hypertrophy changes were confirmed by 3.7-fold increased ANP, 2.7-fold increased of BNP, and 2.1-fold increased Myh7 mRNA expression as compared with Wister Kyoto (WKY) rats (Fig. 1D). Myocardium KLK8 expression was also higher in SHR rats compared with WKY rats (Fig. 1E, *P* < 0.01).

Given that KLK1 is also involved in cardiac hypertrophy, the expression of KLK1 in hypertrophic hearts was also examined. As shown in Fig. 1F,G, KLK1 mRNA and protein levels were decreased in myocardium of TAC rats compared with controls. However, there is no significant change in KLK1 mRNA and protein levels in SHR hearts compared with WKY hearts.

### Intra-cardiac injection of Ad-KLK8 leads to cardiac hypertrophy

**Figure 2:**
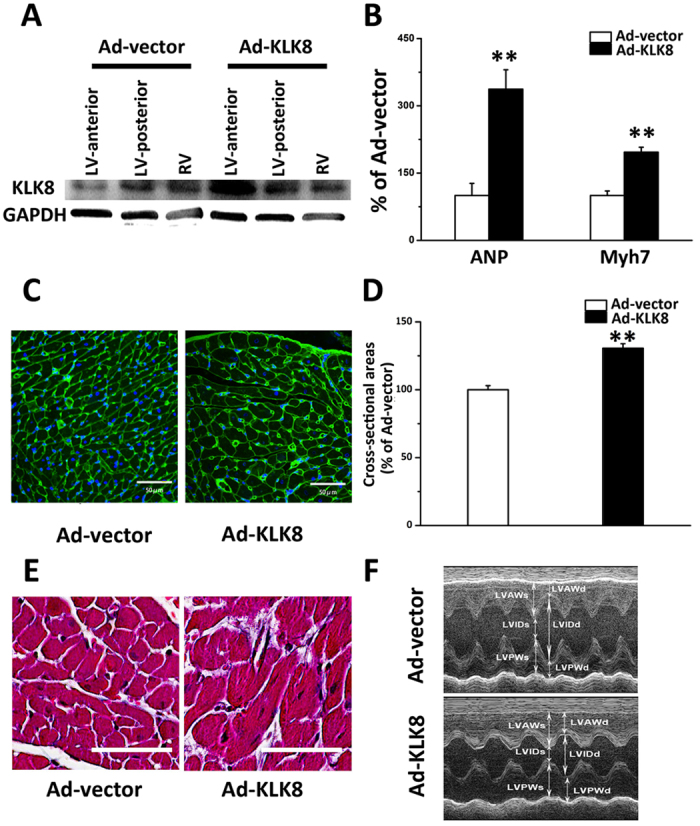
Intra-cardiac injection of Ad-KLK8 leads to cardiac hypertrophy. (**A**) Representative western blot analysis of KLK8 protein expression in the anterior wall of left ventricle (LV), posterior wall of LV and the right ventricle two weeks after intra-cardiac injection of Ad-KLK8 and Ad-control into the anterior wall of LV. B-F, Four weeks after intra-cardiac injection of Ad-KLK8 and Ad-control, experimental animals were used for measurements of cardiac hypertrophic markers (**B**), cross-sectional area (**C,D**), interstitial fibrosis (**E**), as well as echocardiography analysis (**F**). (**B**) mRNA level of cardiac hypertrophic markers (ANP and Myh7) in the anterior wall of LV was determined by quantitative real-time RT-PCR. (**C**) WGA staining was performed on transverse sections of the anterior wall of LV. (**D**) Mean cardiomyocyte cross-sectional area was quantified using the Image-J cell area measurement software. Six rats were analyzed for each group, and 30 to 40 cardiomyocytes were measured per rat (n = 200 cells/group). (**E**) Histological analysis using masson staining. F, Representative M-mode images of rats subjected to intra-cardiac injection of Ad-control or Ad-KLK8. Scale bar: 50 μm. ** vs Ad-vector.

As KLK8 expression was increased in hypertrophy heart tissues, we then investigated whether *in vivo* intra-cardiac adenovirus-mediated KLK8 delivery might lead to cardiac hypertrophy. Two weeks after injection of Ad-KLK8 into the anterior wall of left ventricle (LV), KLK8 expression was confirmed by western blotting. As shown in Fig. 2A, KLK8 expression was robustly increased in the anterior wall of LV tissue, and KLK8 expression was not changed in the posterior wall of LV and the right ventricle. Four weeks after intra-cardiac injection of Ad-vector and Ad-KLK8, the anterior wall of LV tissue was obtained for measurement of cardiac hypertrophy markers and histological analysis. It was found that intra-cardiac KLK8 gene delivery led to a significantly increase in the transcripts of cardiac hypertrophy markers including ANP and Myh7 (Fig. 2B). Analysis of WGA-stained heart sections revealed that the anterior wall of LV tissue obtained from rats injected with Ad-KLK8 have approximately 1.3-fold increase in cross-sectional area of cardiomyocytes as compared with those obtained from rats injected with Ad-control (Fig. 2C,D). Masson’s staining showed that the extent of cardiac fibrosis was comparable in LV tissues obtained from rats injected with Ad-KLK8 or control adenovirus (Fig. 2E).

Echocardiographic analysis was performed to independently measure the diameter of the LV and the thickness of the ventricular walls, as well as LV function 4 weeks after intra-cardiac injection with Ad-KLK8 or control adenovirus. Figure 2F showed representative M-mode echocardiographic images of the hearts. We found that the diameter of the LV was decreased significantly in rats injected with Ad-KLK8 compared with those injected with control adenovirus (Table 1). Notably, the thickness of the anterior LV wall was significantly increased in rats injected with Ad-KLK8 compared with those injected with control adenovirus. The thickness of posterior LV wall was not differed between Ad-KLK8 and control adenovirus mice. LV functions characterized by LV ejection fraction and LV fractional shortening were significantly enhanced in rats injected with Ad-KLK8 compared with those injected with control adenovirus.

### Transgenic overexpression of KLK8 induces cardiac hypertrophy

**Figure 3:**
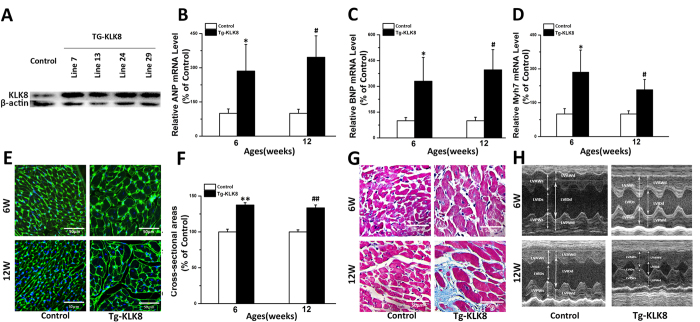
KLK8 transgenic rats show cardiac dysfunction. (**A**) Western blot analysis showed significant increases of KLK8 protein expression in heart homogenates obtained from Tg-KLK8 rats compared with those from control rats. (**B–D**) mRNA level of cardiac hypertrophic markers ANP (**B**), BNP (**C**) and Myh7 (**D**) in hearts from Tg-KLK8 and littermate control rats at the age of 6 and 12 weeks were determined by quantitative real-time RT-PCR. (**E**) WGA staining was performed on transverse sections of the hearts from Tg-KLK8 and littermate control rats at the age of 6 and 12 weeks. (**F**) Mean cardiomyocyte cross-sectional area was quantified using the Image-J cell area measurement software. Six rats were analyzed for each group, and 30 to 40 cardiomyocytes were measured per rat (n = 200 cells/group). (**G**) Histological analysis using masson staining of the hearts from Tg-KLK8 and littermate control rats at the age of 6 and 12 weeks. H, Representative M-mode images of Tg-KLK8 and littermate control rats at the age of 6 and 12 weeks. Scale bar: 50 μm. *P < 0.05, **P < 0.01 vs 6-week old littermate control rats; ^#^P < 0.05, ^##^P < 0.01 vs 12-week old littermate control rats.

To further define the role of KLK8 overexpression *in vivo*, we established KLK8 transgenic rats. In four independent lines, all of the rats were born normally and survived to adulthood and were fertile. KLK8 is overexpressed in the heart in all lines of Tg-KLK8 rats (Fig. 3A). The relative levels of KLK8 expression in the different lines were line No. 7 > 24 > 29 > 13. In this study, we presented the results of experiments in Tg-KLK8 line 7, which showed the highest expression level of KLK8 in the heart. As shown in Fig. 3 and Table 2, the KLK8 transgenic rats developed significant cardiac hypertrophy at both 6-weeks and 12-weeks of age. They showed higher left ventricle weight/body weight (LVW/BW) and heart weight/body weight (HW/BW) ratios than the littermate controls (Table 2). The transcripts of cardiac hypertrophy markers including ANP, BNP and Myh7 (Fig. 3B–D) were also significantly up-regulated in the heart tissues of KLK8 transgenic rats. Quantitative measurement of 6-weeks-old and 12-weeks-old KLK8 transgenic cardiomyocytes using WGA staining revealed 1.37-fold and 1.33-fold increase in cell cross-sectional area as compared to 6-weeks-old and 12-weeks-old control rats, respectively (Fig. 3E,F). Masson’s staining showed that the extent of cardiac fibrosis was comparable in LV tissues obtained from 6-weeks-old KLK8 transgenic rats or 6-weeks-old control rats (Fig. 3G). However, the area of fibrosis seen on Masson’s trichrome staining was significantly increased in LV tissues obtained from 12-weeks-old KLK8 transgenic rats compared with those obtained from 12-weeks-old control rats (Fig. 3G).

### KLK8 transgenic rats display cardiac dysfunction

To evaluate the effect of transgenic overexpression of KLK8 on cardiac function, we first assessed the LV dimensions and functions of 6-weeks-old and 12-weeks-old KLK8 transgenic rats by using M-mode echocardiography. As shown in Table 2, thickening of the LV walls, as well as a decrease in the LV internal diameter in diastole, were shown in KLK8 transgenic rats as compared with the littermate control rats, at both 6-weeks and 12-weeks of age. Notably, both ejection fraction and fractional shortening were increased in 6-weeks-old KLK8 transgenic rats, however were decreased in 12-weeks-old KLK8 transgenic rats, as compared with the littermate control rats.

Functional invasive hemodynamic measurements were performed to confirm the occurrence of echocardiographic alterations in KLK8 transgenic rats (Table 2). There was no significant difference in heart rates between the control and transgenic rats. Peak rates of ventricular contraction (dP/dt_max_) and relaxation (dP/dt_min_) were increased in 6-weeks-old KLK8 transgenic rats, however were decreased in 12-weeks-old KLK8 transgenic rats, as compared with the littermate control rats. These results reflected the progressive cardiac dysfunction in KLK8 transgenic rats.

### KLK8 induces cardiomyocyte hypertrophy and contributes to PE-induced cardiomyocyte hypertrophy *in vitro*

**Figure 4:**
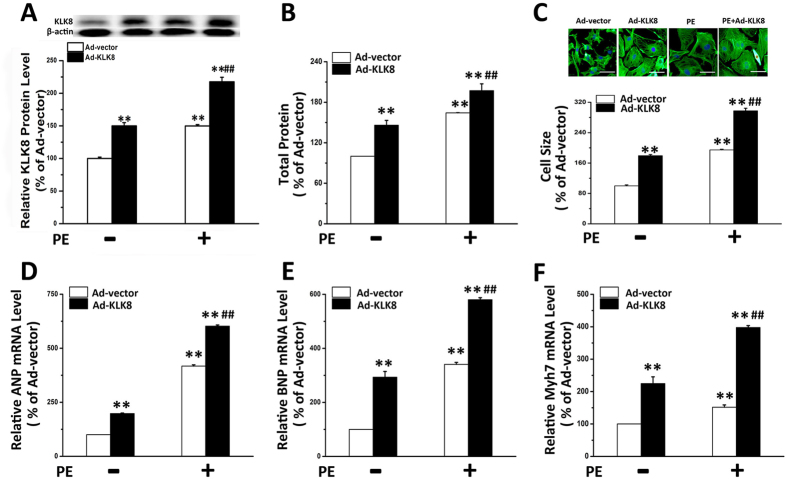
KLK8 induces cardiomyocyte hypertrophy and aggravates PE-induced cardiomyocyte hypertrophy *in vitro*. Primary cultured neonatal cardiomyocytes were infected with KLK8 adenovirus. After incubation for 72 h, cells were treated with PE for another 48 h. (**A**) showed KLK8 expression level in cardiomyocytes treated by KLK8 adenovirus with or without PE. (**B**) total protein content was determined by BCA assay; (**C**) Cardiomyocytes were stained with FITC-conjugated phalloidin as described in “Methods”. One hundred cells from randomly selected fields in three independent expreriments were evaluated for cell size using the Image-Pro Plus cell area measurement software. Representative images are on the top of the corresponding graph. (**D–F**) transcripts of cardiac hypertrophy markers including ANP (**D**), BNP (**E**) and Myh7 (**F**) were determined by quantitative real-time RT-PCR. Values are presented as mean ± SEM (n = 3). Scale bar: 50 μm. **P < 0.01 vs cells treated with Ad-vector; ^##^P < 0.01 vs cells treated with Ad-vector and PE.

To further confirm the role of KLK8 in cardiomyocyte hypertrophy, KLK8 was overexpressed in cultured cardiomyocytes with KLK8 adenovirus. As shown in Fig. 4A, infection of cardiomyocytes with KLK8 adenovirus resulted in KLK8 overexpression. Overexpression of KLK8 *per se* significantly increased cardiomyocyte protein content (Fig. 4B), cell size (Fig. 4C) and transcripts of ANP, BNP and Myh7 (Fig. 4D–F) in cardiomyocytes. We also found that KLK8 expression in cardiomyocytes was significantly induced by PE treatment. Moreover, Ad-KLK8 and PE treatment had an additive effect on KLK8 expression (Fig. 4A). As shown in Fig. 4B–F, KLK8 overexpression substantially aggravated PE-induced cardiomyocyte hypertrophy.

**Figure 5:**
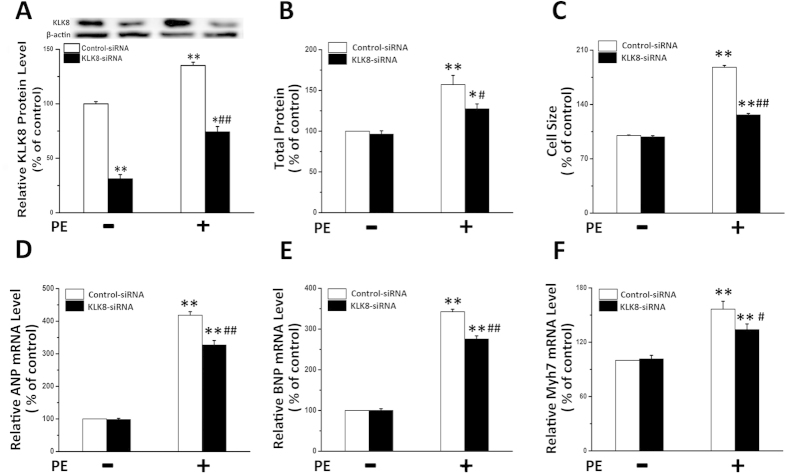
Knockdown of KLK8 inhibits PE-induced cardiomyocyte hypertrophy. Primary cultured neonatal cardiomyocytes were transfected with control siRNA or KLK8 siRNA for 24 h, and then treated with PE for another 48 h. (**A**) showed KLK8 protein expression. (**B**) total protein content was determined by BCA assay; (**C**), Cardiomyocytes were stained with FITC-conjugated phalloidin as described in “Methods”. One hundred cells from randomly selected fields in three independent expreriments were evaluated for cell size using the Image-Pro Plus cell area measurement software. (**D–F**) transcripts of cardiac hypertrophy markers including ANP (**D**), BNP (**E**) and Myh7 (**F**) were determined by quantitative real-time RT-PCR. Values are presented as mean ± SEM (n = 3). *P < 0.05, **P < 0.01 vs cells treated with Control siRNA; ^#^P < 0.05, ^##^P < 0.01 vs cells treated with Control siRNA and PE.

As KLK8 expression is increased in hypertrophy heart tissues, we then investigated whether endogenous KLK8 is involved in cardiomyocyte hypertrophy. As shown in Fig. 5A, transfection of siRNA targeting KLK8 not only resulted in approximately 70% decrease in basal KLK8 protein expression, but also significantly decreased PE-induced KLK8 expression in cardiomyocytes. PE caused an increase in protein content, cell size, and the transcripts of hypertrophy markers including ANP, BNP and Myh7 in cultured cardiomyocytes (Fig. 5B–F). KLK8 siRNA partly reversed the PE-induced hypertrophy as showing decreased protein content, cell size, and the mRNA levels of ANP, BNP and Myh7. KLK8 siRNA didn’t affect the basal levels of above parameters in cultured cardiomyocytes.

### The hypertrophic effect of KLK8 is not via kinin receptors but dependent on its proteolytic activity

**Figure 6:**
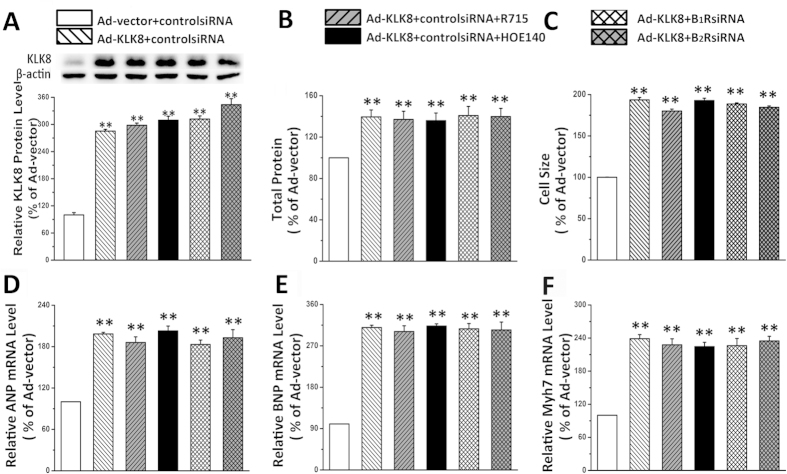
KLK8-induced cardiomyocyte hypertrophy is not dependent on kinin B_1_R and B_2_R. Primary cultured neonatal cardiomyocytes were infected with KLK8 adenovirus, 24 h later control siRNA, kinin B_1_R siRNA or B_2_R siRNA was transfected into the cardiomyocytes. Twenty four hours later, all the media of cells were replaced with fresh media, then kinin B_1_R antagonist R715, or kinin B_2_R antagonist HOE140 was added into the culture media, After incubation for 24 h, cells were treated with PE for another 48 h. (**A**) showed KLK8 protein expression; (**B**) total protein content was determined by BCA assay; (**C**) Cardiomyocytes were stained with FITC-conjugated phalloidin as described in “Methods”. One hundred cells from randomly selected fields in three independent expreriments were evaluated for cell size using the Image-Pro Plus cell area measurement software. (**D–F**) transcripts of cardiac hypertrophy markers including ANP (**D**), BNP (**E**) and Myh7 (**F**) were determined by quantitative real-time RT-PCR. Values are presented as mean ± SEM (n = 3). **P < 0.01 vs cells treated with Ad-vector and control siRNA.

We then investigated the molecular mechanisms responsible for KLK8 induction of hypertrophy. KLK family is supposed to cleave kininogens to release kinin. Kinin is well known to have protective effect in cardiomyocytes by interacting with kinin receptors^1^,^2^. We found that infection of cardiomyocytes with KLK8 adenovirus resulted in a 3.56 ± 0.58 fold increase in the content of bradykinin in culture media. However, neither kinin B_1_R antagonist R715 nor kinin B_2_R antagonist HOE140 reversed the hypertrophic effects of KLK8 (Fig. 6B–F). To further confirm the role of kinin receptors in KLK8 induced cardiomyocytic hypertrophy, we used siRNA approach to knockdown kinin B_1_R and B_2_R (Supplementary Fig. 1A,B). It was found that knockdown of B_1_R or B_2_R could not reverse KLK8-induced hypertrophy (Fig. 6B–F). As shown in Supplementary Fig. 2 and 3, neither antagonists nor knockdown of kinin B_1_/B_2_ receptors reversed the additive effect of Ad-KLK8 and PE on cardiomyocytic hypertrophy.

**Figure 7:**
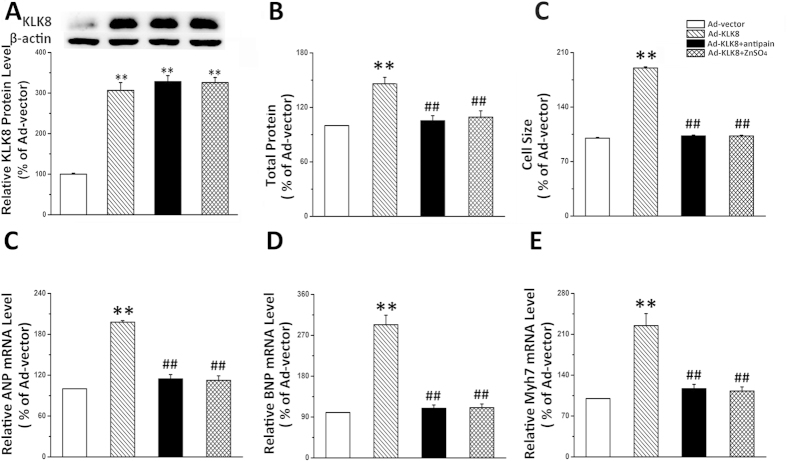
Serine protease inhibitor entirely blocks the hypertrophic effects of KLK8. Primary cultured neonatal cardiomyocytes were infected with KLK8 adenovirus, 24 h later serine protease inhibitor antipain or ZnSO4 was added into the culture media. After incubation for 24 h, cells were treated with PE for another 48 h. (**A**) showed KLK8 protein expression; (**B**) total protein content was determined by BCA assay; (**C**) Cardiomyocytes were stained with FITC-conjugated phalloidin as described in “Methods”. One hundred cells from randomly selected fields in three independent expreriments were evaluated for cell size using the Image-Pro Plus cell area measurement software. (**D–F**) transcripts of cardiac hypertrophy markers including ANP (**D**), BNP (**E**) and Myh7 (**F**) were determined by quantitative real-time RT-PCR. Values are presented as mean ± SEM (n = 3). **P < 0.01 vs cells treated with Ad-vector; ^##^P < 0.01vs cells treated with Ad-KLK8.

As KLK8 is a member of trypsin- and chymotrypsin-like serine proteases, we then investigated whether the hypertrophic effect of KLK8 is through its proteolytic activity. Two serine protease inhibitors, antipain and ZnSO4 were used to block the proteolytic activity of KLK8 as described previously^8^. As shown in Fig. 7, antipain and ZnSO4 entirely blocked the hypertrophic effects of KLK8. In addition, the additive effect of Ad-KLK8 on PE-induced cardiomyocytic hypertrophy was abolished by antipain and ZnSO4 (Supplementary Fig. 4). Taken together, these findings suggest that the hypertrophic effect of KLK8 is dependent on its proteolytic activty

### EGF signaling, PAR1 and PAR2 contribute to the hypertrophic effect of KLK8 *in vitro* and *in vivo*

**Figure 8:**
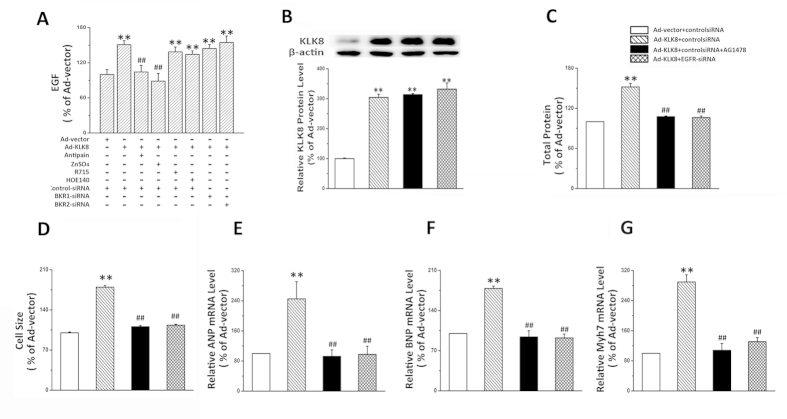
The hypertrophic effect of KLK8 is dependent on EGF signaling. (**A**) Primary cultured neonatal cardiomyocytes were infected with KLK8 adenovirus, 24 h later control siRNA, kinin B_1_R siRNA or B_2_R siRNA was transfected into the cardiomyocytes. Twenty four hours later, all the media of cells were replaced with fresh media, then serine protease inhibitors (antipain or ZnSO4), kinin B_1_R antagonist R715, or kinin B_2_R antagonist HOE140 was added into the culture media, After incubation for 24 h, culture media were collected for determination of EGF. (**B–F**) Cells were infected with KLK8 adenovirus, 24 h later control siRNA or EGFR siRNA was transfected into the cardiomyocytes. Twenty four hours later, all the media of cells were replaced with fresh media, then EGFR antagonist AG1478 was added into the culture media. The hypertrophic effects were detected after additional 48 h of incubation. B showed KLK8 protein expression. Total protein content was determined by BCA assay (**C**). (**D**) Cardiomyocytes were stained with FITC-conjugated phalloidin as described in “Methods”. One hundred cells from randomly selected fields in three independent expreriments were evaluated for cell size using the Image-Pro Plus cell area measurement software. Transcripts of cardiac hypertrophy markers including ANP (**E**), BNP (**F**) and Myh7 (**G**) were determined by quantitative real-time RT-PCR. Values are presented as mean ± SEM (n = 3). **P < 0.01 vs cells treated with Ad-vector and control siRNA; ^##^P < 0.01 vs cells treated with Ad-KLK8 and control siRNA.

KLKs have the ability to hydrolyze a variety of substrates such as pro-epidermal growth factor (pro-EGF). It is well known that EGF induces hypertrophy in various cell types^24^,^25^. Thus, we investigated whether the hypertrophic effect of KLK8 was through releasing EGF. As shown in Fig. 8A, it was found that overexpression of KLK8 in cardiomycytes significantly increased the EGF level in the culture media. This effect was completely abolished by serine protease inhibitors antipain and ZnSO4. Neither pharmacological antagonists nor siRNAs of kinin B1 and B2 receptors affected EGF level in KLK8 overexpressed cells.

To confirm EGF is involved in hypertrophic effect of KLK8, the effects of the antagonist of EGF receptor and siRNA targeting EGF receptor (EGFR) on KLK8-induced cardiomyocyte hypertrophy were then investigated. As shown in Fig. 8C–G, EGF receptor antagonist blocked hypertrophic effects of KLK8. In addition, we also used EGFR siRNA, which resulted in 70% reduction of EGFR (Supplementary Fig. 1C). It was found that knockdown of EGFR also significantly attenuated KLK8-induced hypertrophy.

**Figure 9:**
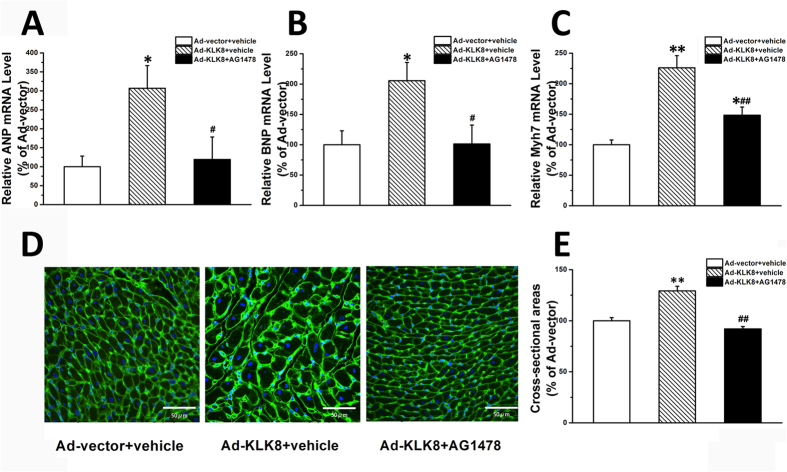
Administration of EGFR antagonist significantly attenuated the hypertrophic effects of intra-cardiac Ad-KLK8 gene delivery *in vivo*. Adenovirus particles containing KLK8 or control vector were administered to rats by intra-cardiac injection into the anterior wall of left ventricular. Rats injected by Ad-KLK8 were then treated by selective EGFR antagonist AG1478 (3 mg/kg/day, i.p.). Two weeks after intra-cardiac injection of Ad-KLK8 and Ad-control, experimental animals were used for measurements of cardiac hypertrophic markers (**A–C**) and cross-sectional area (**D,E**). (**A–C**) mRNA level of cardiac hypertrophic markers ANP (**A**), BNP (**B**) and Myh7(**C**) in the anterior wall of LV was determined by quantitative real-time RT-PCR. (**D**) WGA staining was performed on transverse sections of the anterior wall of LV. (**E**) Mean cardiomyocyte cross-sectional area was quantified using the Image-J cell area measurement software. Six rats were analyzed for each group, and 30 to 40 cardiomyocytes were measured per rat (n = 200 cells/group). Scale bar: 50 μm. *P < 0.05, **P < 0.01 vs Ad-vector; ^#^P < 0.05, ^##^P < 0.01 vs Ad-KLK8.

We next examined whether EGF signaling contributed to the hypertrophic effect of KLK8 *in vivo*. As shown in Fig. 9, it was found that administration of EGFR antagonist (3 mg/kg) significantly attenuated the hypertrophic effects of intra-cardiac Ad-KLK8 gene delivery, as evidenced by the decreases in the transcripts of cardiac hypertrophy markers (Fig. 9A–C) and cross-sectional area of cardiomyocytes (Fig. 9D,E) as compared with those obtained from rats injected with Ad-KLK8 alone.

**Figure 10:**
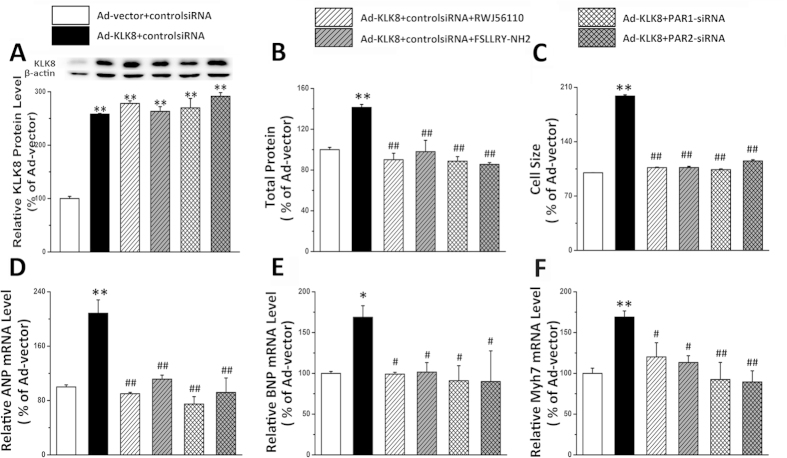
Both PAR1 and PAR2 are involved in the hypertrophic effect of KLK8. Primary cultured neonatal cardiomyocytes were infected with KLK8 adenovirus, 24 h later control siRNA, PAR1 siRNA or PAR2 siRNA was transfected into the cardiomyocytes. Twenty four hours later, all the media of cells were replaced with fresh media, then PAR1 antagonist RWJ56110, or PAR2 antagonist FSLLRY-NH2 was added into the culture media. After incubation for 24 h, cells were treated with PE for another 48 h. (**A**) showed KLK8 protein expression. (**B**) total protein content was determined by BCA assay; (**C**) Cardiomyocytes were stained with FITC-conjugated phalloidin as described in “Methods”. One hundred cells from randomly selected fields in three independent expreriments were evaluated for cell size using the Image-Pro Plus cell area measurement software. (**D–F**) transcripts of cardiac hypertrophy markers including ANP (**D**), BNP (**E**) and Myh7 (**F**) were determined by quantitative real-time RT-PCR. Values are presented as mean ± SEM (n = 3). *P < 0.05, **P < 0.01 vs cells treated with Ad-vector and control siRNA; ^#^P < 0.05, ^##^P < 0.01vs cells treated with Ad-KLK8 and control siRNA.

KLKs have been demonstrated to regulate cell signaling by cleaving and activating the protease-activated receptors (PARs), which have been implicated in many cellular effects including hypertrophy development^26^,^27^. Thus, we investigated whether PARs contribute to the hypertrophic effect of KLK8. As shown in Fig. 10, both PAR1 antagonists RWJ56110 and PAR2 antagonist FSLLRY-NH2 significantly attenuated the hypertrophic effects of KLK8. In addition, we also used siRNAs targeting PAR1 and PAR2, which resulted in 80% and 85% reduction of PAR1 and PAR2, respectively (Supplementary Fig. 1D,E). It was found that knockdown of either PAR1 or PAR2 also blocked KLK8-induced hypertrophy (Fig. 10).

**Figure 11:**
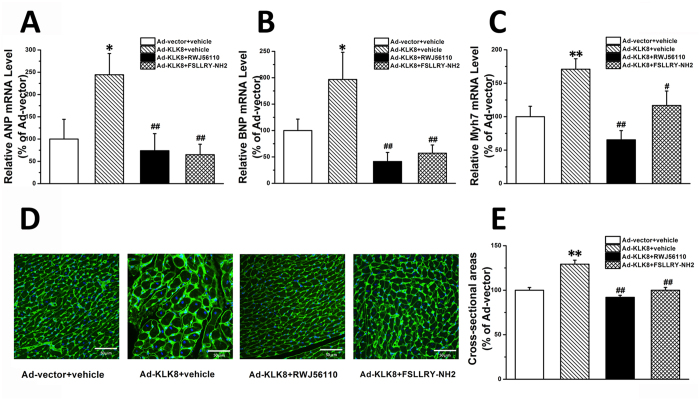
Administration of PAR1 or PAR2 antagonist significantly attenuated the hypertrophic effects of intra-cardiac Ad-KLK8 gene delivery *in vivo*. Adenovirus particles containing KLK8 or control vector were administered to rats by intra-cardiac injection into the anterior wall of left ventricular. Rats injected by Ad-KLK8 were then treated by selective PAR1 antagonist RWJ56110 (1 mg/kg/day, i.p.) or PAR2 antagonist FSLLRY-NH2 (1 mg/kg/day, i.p.). Two weeks after intra-cardiac injection of Ad-KLK8 and Ad-control, experimental animals were used for measurements of cardiac hypertrophic markers (**A–C**) and cross-sectional area (**D,E**). (**A–C**) mRNA level of cardiac hypertrophic markers ANP (**A**), BNP (**B**) and Myh7(**C**) in the anterior wall of LV was determined by quantitative real-time RT-PCR. (**D**) WGA staining was performed on transverse sections of the anterior wall of LV. (**E**) Mean cardiomyocyte cross-sectional area was quantified using the Image-J cell area measurement software. Six rats were analyzed for each group, and 30 to 40 cardiomyocytes were measured per rat (n = 200 cells/group). Scale bar: 50 μm. *P < 0.05, **P < 0.01 vs Ad-vector; ^#^P < 0.05, ^##^P < 0.01 vs Ad-KLK8.

In the *in vivo* studies, it was found that administration of PAR1 antagonists RWJ56110 (1 mg/kg) and PAR2 antagonist FSLLRY-NH2 (1 mg/kg) significantly attenuated the hypertrophic effects of intra-cardiac Ad-KLK8 gene delivery, as evidenced by the decreases in the transcripts of cardiac hypertrophy markers (Fig. 11A–C) and cross-sectional area of cardiomyocytes (Fig. 11D,E) as compared with those obtained from rats injected with Ad-KLK8 alone.

**Figure 12:**
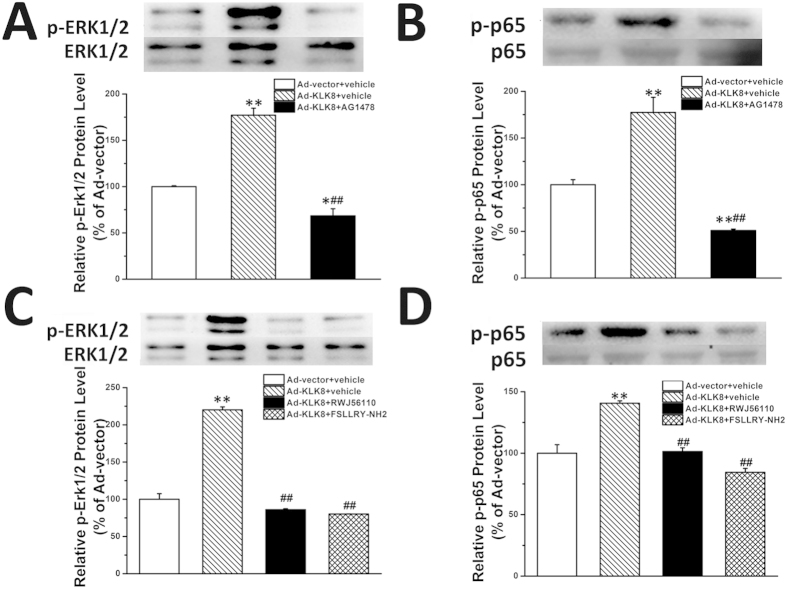
Intra-cardiac injection of KLK8 resulted in significant increases of phosphor-ERK1/2 and phosphor-NFκB levels, which were attenuated by administration of EGFR antagonist, PAR1 antagonist, or PAR2 antagonist. Adenovirus particles containing KLK8 or control vector were administered to rats by intra-cardiac injection into the anterior wall of left ventricular. Rats injected by Ad-KLK8 were then treated by selective EGFR antagonist AG1478 (3 mg/kg/day, i.p.), PAR1 antagonist RWJ56110 (1 mg/kg/day, i.p.), or PAR2 antagonist FSLLRY-NH2 (1 mg/kg/day, i.p.). Two weeks after intra-cardiac injection of Ad-KLK8 and Ad-control, levels of phosphor-ERK1/2 and phosphor-NFκB p65 subunit in the anterior wall of LV was determined by Western blot analysis. *P < 0.05, **P < 0.01 vs Ad-vector; ^##^P < 0.01 vs Ad-KLK8.

Previous studies have demonstrated that EGFR and PARs activate some common effector pathways such as PLC, ERK1/2, p38 MAPK and Akt^28^,^29^. Among these signaling molecules, ERK1/2 is known to be involved in cardiac hypertrophy-induced by both EGFR and PARs. Mechanically, the ERK1/2 pathway induces cardiac hypertrophy may, at least in part, by enhancing the transcriptional activity of NFκB, an important hypertrophic transcription factor^30^. Thus, we next investigated whether KLK8 activated ERK1/2 and NFκB via EGFR and PAR *in vivo*. As shown in Fig. 12, we found that intra-cardiac injection of KLK8 resulted in significant increases of phosphor-ERK1/2 and phosphor-NFκB p65 subunit levels, which were attenuated by administration of EGFR antagonist, PAR1 antagonist, or PAR2 antagonist.

## Discussion

The present study demonstrates for the first time that KLK8 is expressed in cardiomyocytes. Furthermore, we provide the evidence that KLK8 induces hypertrophy in cardiomyocytes both *in vivo* and *in vitro*.

Previous studies have demonstrated that KLK1 reduces cardiac hypertrophy and fibrosis in various cardiac hypertrophy models^31^,^32^,^33^,^34^,^35^. KLK1 acts mainly on low-molecular weight kininogen to produce kinin peptides, which exert anti-hypertrophic effects mainly via kinin B_2_R^31^,^34^. Kinins are continuously released during cardiac hypoxia, ischemia and myocardial infarction^36^. Cardiac KLK1-kinin system is implicated to be a prime mediator in protecting the heart in hypertrophic conditions^37^. It is known that kinin peptides consist of bradykinin and kallidin in humans or bradykinin and the kallidin-like peptide in rodents^38^. All of these peptides could interact with kinin B_1_R and B_2_R. In addition, it has been shown that, in kninogen deficient rats, KLK1 can reduce cardiomyocytic apoptosis and improve cardiac performance of infarcted hearts via kinin B_2_R, suggesting that KLK1 may release “kinin-like peptides” not from kininogen^39^. In the present study, we found that KLK8 could release bradykinin in cardiomyocytes, meanwhile significantly promote cardiac hypertrophy. These differences in KLK1 and KLK8 effects suggest that KLK1 and KLK8 may release different peptides, which have different effects on hypertrophy.

Besides kininogens, a variety of substrates can be degraded by KLK family serine proteases. For instance, both KLK1 and KLK8 degrade extracellular matrix proteins including collagen and fibronectin^1^,^2^,^8^,^9^. Guillon-Munos A *et al.*^40^ have indicated that KLK12 induces hydrolysis of matricellular proteins of the CCN (cyr61, ctgf, nov) family which contributes to the bioavailability and/or activity of several growth factors including VEGF, BMP2, TGF-β1 and FGF-2. More recently, Sanchez WY *et al.*^41^ showed that selective cleavage of human sex hormone-binding globulin (SHBG) by active KLK4 and KLK14 affects androgen action in LNCaP prostate cancer cells. In the present study, we found that the hypertrophic effect of KLK8 was entirely abolished by inhibition of KLK8 serine protease activity, but was not affected by either pharmacological blockade or siRNA-mediated knock-down of kinin B1R and B2R. These results indicate that KLK8 induction of hypertrophy be not through releasing kinin peptides but is dependent on its serine protease activity. Notably, a limitation of this study is that KLK8 proteolytic activity was not assessed in KLK8 overexpression cells since it is difficult to evaluate KLK8 activity on the cellular level. To date, gelatin zymography is the most usually used method to determine proteolytic activity of KLK family members including KLK8^42^. As gelatin is the common substrate for KLK family members^43^, this method is not specific for KLK8 and mainly used to assess recombinant KLK8 activity *in vitro*. However, as mentioned, we showed that the inhibitors of serine protease reversed hypertrophy induced by KLK8 overexpression.

EGF plays an important role in promoting hypertrophic responses in cardiac pathologies^24^,^25^. It has been shown that active EGF signaling pathway can be induced by KLK family members in a variety of cell types. For example, KLK1 cleaved pro-epidermal growth factor (EGF) into mature EGF, which finally activated EGF receptor (EGFR) signaling pathway and led to hypertrophy of primary cultured tracheal submucosal gland cells^10^. More recently, Abdallah *et al.*^44^ demonstrated in primary vascular smooth muscle cells that plasma kallikrein was capable of stimulating a disintegrin and metalloprotease (ADAM) 17 activity, and leading sequentially to the release of the endogenous EGF receptor ligand amphiregulin which contributed to the transactivation of EGF receptor and ERK1/2 activation. We found that overexpression of KLK8 led to an increase in EGF, which could be abolished by serine protease inhibitors. In addition, blockade of EGF receptor reversed KLK8-induced hypertrophy both *in vitro* and *in vivo*. These results suggest that KLK8 promote cardiomyocyte hypertrophy via activation of EGF signaling pathway.

PARs are well-known to be activated by thrombin or other coagulation or inflammatory proteases formed at sites of tissue injury^45^. PAR1 activation triggers a range of signaling events in cardiomyocytes that lead to changes in electrical/mechanical function^45^. Cardiomyocyte-specific overexpression of PAR1 in mice induces eccentric hypertrophy and dilated cardiomyopathy^27^. As for PAR-2, it activates a spectrum of biochemical and functional responses that largely mimic cardiomyocyte activation by PAR-1 (including PLC, ERK, p38-MAPK, increased [Ca^2+^]i, enhanced spontaneous automaticity, and elongated/dilated hypertrophy) in cardiomyocytes^46^. KLKs are recently known to regulate cell function by cleaving and activating members of PAR family. For example, KLK4 and KLK14 mediate PAR1 and PAR2 activation in colon cancer cells, respectively^47^,^48^. KLK6 is an activator of PAR1/2 in neurons and astrocytes^49^. Whether KLK8 activates PAR1/2 signaling remains unknown. The present study found that both PAR1/2 inhibitor and PAR1/2 knockdown significantly reversed KLK8-induced hypertrophy in cardiomyocytes. In addition, administration of PAR1 or PAR2 inhibitor significantly attenuated the hypertrophic effects of intra-cardiac Ad-KLK8 gene delivery. These data provided convincing evidence that PAR1/2 activation contributed to KLK8-induced cardiac hypertrophy. However, the limitation was that we didn’t perform biochemical experiments to investigate how KLK8 cleaved and activated PAR1 and PAR2 in cardiomyocytes. The precise mechanisms involved in PAR1/2 activation by KLK8 should be considered in future studies.

EGFR and PARs are known to activate some common effector pathways such as PLC, ERK1/2, p38 MAPK and Akt^28^,^29^. Among these signaling molecules, ERK1/2 is known to be involved in cardiac hypertrophy-induced by both EGFR and PARs. Mechanically, the ERK1/2 pathway induces cardiac hypertrophy may, at least in part, by enhancing the transcriptional activity of NFκB^30^. The present study found that intra-cardiac injection of KLK8 resulted in significant increases of phosphor-ERK1/2 and phosphor-NFκB levels, which were attenuated by administration of EGFR antagonist, PAR1 antagonist, or PAR2 antagonist. Taken together, these results suggest that ERK1/2-NFκB signaling pathway may be the downstream effector of EGFR and PAR1/2, and play a critical role in the hypertrophic effect of KLK8.

Cardiac ryanodine receptor (RyR2) macromolecular complex, a large ion channel composed of four 560-kDa RyR subunits and four 12.6-kDa FK506 binding proteins (FKBP12.6, also known as calstabin2), is a major determinant of intracellular Ca^2+^ release in cardiomyocytes and required for excitation-contraction coupling^50^. Growing evidence indicates that disturbances of Ca^2+^ dynamics due to dysfunction of RyR2 macromolecular complex results in several cardiac disorders including cardiac hypertrophy and heart failure^50^. For example, recent studies show that genetic deletion or dysfunction of calstabin2 can lead to cardiac hypertrophy and dysfunction^51^. Furthermore, previous reports indicated that calstabin1 (also known as FKBP12), which shares 85% sequence identity with calstabin2, can form an endogenous inhibitor of EGFR phosphorylation directly involved in the control of cellular EGFR activity^52^. Thus, it is of interest to investigate whether calstabin2 contributes to KLK8-induced cardiac hypertrophy and dysfunction through modulating EGFR activity.

It is well-recognized that intrinsic cardiac aging leads to structural and functional deteriorations of the heart in elderly individuals. Cardiac hypertrophy is a hallmark of cardiac aging^53^. Accumulating evidence shows that aging results in an increase in the prevalence of left ventricular hypertrophy, a decline in diastolic function, and relatively preserved systolic function at rest but a decline in exercise capacity^51^,^53^. Recent studies have shown the involvements of multiple molecular mechanisms in the development of cardiac aging. In particular, an imbalance of synthesis and degradation of extracellular matrix proteins has been implicated in the pathogenesis of aging-associated cardiac hypertrophy^54^. Since KLK family members have been observed to be involved in cardiac hypertrophy through modulating ECM synthesis and degradation, whether KLK8 plays a role in aging-associated cardiac hypertrophy merits further investigation.

As a selective α1-adrenoceptor agonist, phenylephrine has been reported to mediate cardiac hypertrophy and aortic contractile responses^55^. The present study found that PE could promote expression of KLK8 in neonatal myocytes. KLK8 knockdown partly reversed the PE-induced hypertrophy, whereas PE and KLK8 overexpression have an additive effect on cardiomyocyte hypertrophy. In the presence of two protease inhibitors, antipain and ZnSO4, cardiomyocytes treated with PE plus KLK8 overexpression showed no significant differences on hypertrophic markers as compared with cells treated with PE only, indicating that protease inhibitors abolished the additive effect of KLK8 overexpression on PE-induced hypertrophy. Taken together, these results let us suggest that the involvement of KLK8 in PE-induced cardiomyocytic hypertrophy might dependent on the serine proteolytic activity of KLK8.

As mentioned before, KLK proteins are secreted as enzymatically inactive proKLKs, which are further processed by other KLKs or other proteases to become active enzymes^1^,^2^. It has been demonstrated that KLK family members can co-localize in same tissue and biological fluid and can be activated by each other through an orchestrated proteolytic cascades^56^,^57^. For example, KLK5 autoactivates, and then can activate the pro-forms of KLK1, KLK6, KLK8, and KLK11 etc. More recently, it has been reported that the active KLK8 could target pro-KLK1 and pro-KLK11^42^. In the present study, it is uncertain whether KLK8 exerts its functions through activating other KLKs, such as KLK1, or KLK1 exerts its function through KLK8. However, our data indicate that at least two KLK family member are co-expressed in cardiomyocytes and have effects on hypertrophy.

We found that KLK1 and KLK8 showed different expression pattern in hypertrophic myocardium. In TAC-induced hypertrophy, KLK8 was up-regulated whilst KLK1 was down-regulated. Given that KLK8 induces hypertrophy whereas KLK1 inhibits hypertrophy, upregulation of KLK8 and downregulation of KLK1 would exacerbate the progress of hypertrophy. Our data that there was no significant difference in KLK1 expression in myocardium between SHR and WKY rats are not consistent with other studies where it has been demonstrated that cardiac KLK1 activity is significantly decreased in SHR rats as compared to WKY rats^58^. However, their study did not show the protein level of KLK1. It is known that serine protease activity does not always parallel its protein level. Nevertheless, the differences in KLK1 and KLK8 expression pattern in cardiac hypertrophy suggest that KLK1 and KLK8 might play different roles in this cardiac disorder. The molecular mechanisms responsible for modulating KLK1 and KLK8 levels in the pathogenesis of hypertrophy remain to be investigated.

## Conclusion

The present study demonstrates that KLK8, a new member of KLK family, is expressed in cardiomycytes and can induce cardiac hypertrophy. KLK8 promotes hypertrophy through EGF and PAR1/2 signaling-dependent and kinin receptor-independent signaling mechanisms. It is implied that different KLK members subtle regulate cardiac function and remodeling.

## Methods

### Animals

Male Sprague-Dawley rats (8-week-old), SHR rats (16-week-old) and WKY rats (16-week-old) were obtained from Shanghai SLAC Laboratory Animal Co (Shanghai, China) and housed at controlled room temperature with free access to food and water under a natural day/night cycle. All animal protocols were approved by the Ethical Committee of Experimental Animals of Second Military Medical University, and confirmed to the principal in the Guide for the Care and Use of Laboratory Animals published by the United States National Institute of Health (NIH publication No. 85-23, revised 1996).

### Transverse aortic constriction (TAC)

Left ventricular hypertrophy was induced by abdominal TAC in male SD rats as described previously^34^. Briefly, the aorta was exposed through a midline abdominal incision. A blunt 21-gauge needle was then placed adjacent to the abdominal aorta above the renal arteries. A ligature was tightened around the aorta and the needle was removed after ligation. Sham-operated rats underwent the same surgical procedure without the actual ligation. Rats were sacrificed at 56 days after aortic constriction.

### Histology and Immunohistology

Paraffin heart sections (5 μm) were stained with KLK8 (sc-67666, Santa Cruz Biotechnology) or Masson trichrome (to examine interstitial fibrosis) as previously described^59^. To confirm the specificity of primary antibody of KLK8, pre-absorption of the primary antibody with a 10-fold excess of the blocking peptides sc-67666 P (Santa Cruz Biotechnology, Inc.) was performed as negative controls. To measure myocyte cross-sectional area, we used fluorescence-tagged wheat germ agglutinin (WGA) staining (5.0 μg/mL; with samples incubated in the dark for 10 minutes at 37 °C). Images were recorded at 494-nm excitation and 518-nm emission and were evaluated with ImageJ^60^.

### Construction of recombinant adenovirus carrying KLK8

KLK8 adenovirus was generated by using the AdEasyTM adenoviral vector system (Stratagene, La Jolla, CA, USA). Rat KLK8 recombinant adenoviruses were finally harvested, purified, and tittered by standard methods^61^. An empty adenoviral construct used at the same titer served as control. The details are described in the Supplementary materials and methods. The overexpression of KLK8 was achieved by infecting cultured neonatal rat ventricular cardiomyocytes with the recombinant adenovirus (multiplicity of infection [MOI] = 10). For *in vivo* gene delivery, 5 × 10^11^ adenovirus particles containing KLK8 or control vector were administered by direct intra-cardiac injection into the anterior wall of left ventricular (5 sites, 50 μl/site), using a syringe with a 30-gauge needle^62^,^63^.

### Construction of the KLK8 Transgenic rats

F0 transgenic Sprague-Dawley rat embryos were generated by pronuclear injection of the expression vector containing the rat KLK8 cDNA by Cyagen Biosciences Inc. (Guangzhou, China). Founders and the offspring were maintained on a 12 h light/dark cycle and fed standard rat chow ad libitum in the Animal Research Center of Second Military Medical University. Genotyping of KLK8 transgenic rats was determined by PCR screening of genomic DNA extracted from rat-tail biopsies using two primer pairs called CMV-F/KLK8-R and KLK8-F/IRES-R. Primer sequences were shown in Supplementary Table 2.

### *In vivo* hemodynamic measurements

Hemodynamic parameters and cardiac function were measured in 6-week and 12-week ages of Tg-KLK8 rats and the wild type littermates. Briefly, rats underwent left ventricular catheterization via left common carotid artery. Hemodynamic variables were measured with a pressure transducer and recorded on a BL-420 S Biologic Function Experiment system (Taimeng technology, Chengdu, China).

### Echocardiography

Echocardiography was performed using a 7.5-MHz phased-array transducer (Acuson Sequoia 256, Siemens, Mountain View, CA) at 28 days after intra-myocardial KLK8 gene delivery as previously described^64^. Left-ventricular end-diastolic and -systolic internal diameter (LVIDd and LVIDs, respectively), left-ventricular end-diastolic and -systolic anterior wall thickness (LVAWd and LVAWs, respectively) and left-ventricular end-diastolic and -systolic posterior wall thickness (LVPWd and LVPWs, respectively) were measured. The cardiac systolic function was indicated by fractional shortening (FS) and ejection fraction (EF). All measurements represented the mean values of the signals from three consecutive cardiac cycles and were carried out by two experienced technicians who were unaware of the identities of the respective experimental groups.

### Preparation of rat neonatal cardiomyocytes culture

Ventricle myocytes were isolated from rats that were up to 3 days old, and were isolated and cultured as described previously^65^. Briefly, ventricle tissues were minced and serially digested. Cell pellets were resuspended and placed in culture dishes at 37 °C for 1 hour to allow selective attachment of nonmyocytes. Cardiomyocyte-enriched fraction were then seeded at a density of 1 × 10^5^ cells/cm^2^ and cultured in DMEM containing 15 mmol/L HEPES, 10% FBS, 0.1 mmol/L bromodeoxyuridine (BrdU), and antibiotics (100 U/mL penicillin and 100 mg/mL streptomycin) for 48 hours. The culture medium was then exchanged for serum-free DMEM containing the same additives with the exception of BrdU. The details are described in the Supplementary materials and methods.

### RNA interference

The small interfering RNA (siRNA) for KLK8, B_1_R, B_2_R, ERGR, PAR1, PAR2 were designed and synthesized by GenePharma Corporation (Shanghai, China). Transfection of siRNA was performed by using siPORT NeoFx transfection agent (Ambion , Austin, TX) according to the instructions of the manufacturer. The details are described in the Supplementary materials and methods.

### Real-Time RT-PCR

Total RNA from heart tissue or cardiomyocytes was extracted and then reverse transcribed to generate cDNA. Quantitative real-time PCR was carried out using SYBRGreen (F Hoffmann-La Roche Ltd, Basel, Switzerland) as detection dye. The housekeeping gene glyceraldehyde-3-phosphate dehydrogenase (GAPDH) was measured for each sample as an internal control for sample loading and normalization. The sequences of PCR products ascertain that their identities matched to the known sequences of rat KLK8, ANP, BNP and Myh7. The details are described in the Supplementary materials and methods.

### Western blot analysis

Proteins of rat heart tissues and cardiomyocytes were extracted using cold T-Per and M-Per lysis buffer (Pierce Biotechnology), respectively. Protein samples were separated by SDS-PAGE and subsequently transferred to nitrocellulose membranes. All antibodies were purchased from Santa Cruz Biotechnology (Santa Cruz Biotechnology, Inc. Santa Cruz, CA). The details are described in the Supplementary materials and methods.

### Measurement of cell surface area

For cell surface area measurement, cardiomyocytes were plated at a density of 5 × 10^4^ cells/3 cm dish to obtain individually plated cells. At the end of the treatment period, the cardiomyocytes were fixed in PBS plus 4% formaldehyde. After permeabilization with 0.1% Triton X-100 in PBS, fixed cells were incubated 3 h at room temperature with FITC-conjugated phalloidin (1:100, sigma) in PBS containing 1% BSA to visualize myocyte sarcomeres (F-actin). Images were acquired using an inverted Olympus fluorescence microscope. Cell images from six randomly chosen fields (40×objective), representing four separate experiments, were measured using the Image-Pro Plus cell area measurement software, and the percentage of mean cell surface area normalized to control group were calculated. Only cells lying completely within the field were quantified.

### Measurement of bradykinin and EGF

The amounts of bradykinin and EGF in the incubation medium of primary cultured cardiomyocytes were assayed using the commercially available EIA kit (Phoenix Pharmaceuticals, Belmont, CA) and ELISA kit (Peprotech, Rocky Hill, NJ), respectively.

### Treatment with selective inhibitors antagonists of EGFR, PAR1 or PAR2 *in vivo*

Adenovirus particles containing KLK8 or control vector were administered to rats by intra-cardiac injection into the anterior wall of left ventricular. Rats injected by Ad-KLK8 were then treated by selective EGFR antagonist AG1478 (3 mg/kg/day, i.p.), PAR1 antagonist RWJ56110 (1 mg/kg/day, i.p.), or PAR2 antagonist FSLLRY-NH2 (1 mg/kg/day, i.p.) for 2 weeks.

### Statistical analysis

All data were expressed as means ± SEM. For illustrative purposes, some results are presented as the mean percent control ± SEM. When comparing multiple groups, one-way or two-way ANOVA was performed and when significant (p < 0.05), comparisons between each groups were conducted using the Student-Newman-Keuls test. SPSS 13.0 statistical software was used for data analysis. P < 0.05 was considered statistically significant.

## Additional Information

**How to cite this article**: Cao, B. *et al.* Kallikrein-related peptidase 8 is expressed in myocardium and induces cardiac hypertrophy. *Sci. Rep.* **6**, 20024; doi: 10.1038/srep20024 (2016).

## Supplementary Material

Supplementary Information

## Figures and Tables

**Table 1:** Results of echocardiography.

	Ad-vector (n = 6)	Ad-klk8 (n = 6)
LVAWs (mm)	3.02 ± 0.10	3.82 ± 0.07**
LVAWd (mm)	1.79 ± 0.097	2.33 ± 0.12**
LVPWs (mm)	3.10 ± 0.15	3.42 ± 0.43
LVPWd (mm)	2.09 ± 0.06	2.22 ± 0.12
LVIDs (mm)	3.38 ± 0.31	1.87 ± 0.44*
LVIDd (mm)	6.94 ± 0.35	5.58 ± 0.24**
%EF	81.09 ± 2.52	96.95 ± 0.64**
%FS	51.34 ± 2.84	76.86 ± 2.29**

*p < 0.05, **p < 0.01 vs Ad-vector. LVAWs, left-ventricular end-systolic anterior wall thickness; LVAWd left-ventricular end-diastolic anterior wall thickness; LVPWs, left-ventricular end-systolic posterior wall thickness; LVPWd, left-ventricular end-diastolic posterior wall thickness; LVIDs, left-ventricular end-systolic internal diameter; LVIDd, left-ventricular end-diastolic internal diameter; EF, ejection fraction; FS, fractional shortening.

**Table 2:** Hemodynamic and echocardiographic parameters in control and Tg-KLK8 rats

	6-week old Control Tg-KLK8	12-week old Control Tg-KLK8
N	6 6	7 7
LVW/BW (mg/g)	2.41 ± 0.05 3.02 ± 0.09**	2.00 ± 0.09 2.44 ± 0.12^#^
HW/BW (mg/g)	3.25 ± 0.04 3.75 ± 0.20*	2.91 ± 0.09 3.29 ± 0.12^#^
LVAWs (mm)	2.46 ± 0.13 3.27 ± 0.14**	2.63 ± 0.09 3.25 ± 0.2^#^
LVAWd (mm)	1.41 ± 0.08 1.84 ± 0.11**	1.40 ± 0.08 1.93 ± 0.11^##^
LVPWs (mm)	2.64 ± 0.15 3.22 ± 0.14*	2.91 ± 0.14 3.48 ± 0.18^#^
LVPWd (mm)	1.61 ± 0.08 1.87 ± 0.20	1.41 ± 0.10 1.87 ± 0.15^#^
LVIDs (mm)	1.90 ± 0.35 1.75 ± 0.10	2.08 ± 0.19 1.94 ± 0.12
LVIDd (mm)	5.89 ± 0.32 4.70 ± 0.08**	5.54 ± 0.11 4.95 ± 0.18^#^
%EF	78.14 ± 3.62 89.83 ± 3.09*	88.32 ± 1.57 78.19 ± 3.29^#^
%FS	48.37 ± 3.71 62.34 ± 4.17*	59.46 ± 2.38 48.17 ± 3.16^#^
Heart rate, bpm	407 ± 21 437 ± 26	387 ± 11 373 ± 14
dP/dt_max_, mmHg/s	5260 ± 199 6114 ± 239*	6201 ± 592 3782 ± 342^##^
dP/dt_min_, mmHg/s	4171 ± 300 5496 ± 306*	5542 ± 538 3492 ± 364^##^

*p < 0.05, **p < 0.01 vs 6-week old littermate control rats; ^#^p < 0.05, ^##^p < 0.01 vs 12-week old littermate control rats. bpm, beats per minute; dP/dt_max_, maximum first derivative of pressure; dP/dt_min_, minimum first derivative of pressure; LVW, left ventricle weight; BW, body weight; HW, heart weight; LVAWs, left-ventricular end-systolic anterior wall thickness; LVAWd left-ventricular end-diastolic anterior wall thickness; LVPWs, left-ventricular end-systolic posterior wall thickness; LVPWd, left-ventricular end-diastolic posterior wall thickness; LVIDs, left-ventricular end-systolic internal diameter; LVIDd, left-ventricular end-diastolic internal diameter; EF, ejection fraction; FS, fractional shortening.
