# Erratum: Kallikrein-related peptidase 8 is expressed in myocardium and induces cardiac hypertrophy

**DOI:** 10.1038/srep25038

**Published:** 2016-04-29

**Authors:** Buqing Cao, Qing Yu, Wei Zhao, Zhiping Tang, Binhai Cong, Jiankui Du, Jianqiang Lu, Xiaoyan Zhu, Xin Ni

Scientific Reports 6: Article number: 20024; 10.1038/srep20024 published online: 01292016; updated: 04292016.

The original version of this Article contained a typographical error in the spelling of the author Binhai Cong, which was incorrectly given as Binghai Cong. This has now been corrected in the PDF and HTML versions of the Article, as well as the Supplementary Information file that now accompanies the Article.

In the original HTML version of this Article there was a typographical error in the volume number ‘6’ which was incorrectly given as ‘7’. This has now been corrected.

