# Supplementary material for: Kallikrein-related peptidase 8 is expressed in myocardium and induces cardiac hypertrophy
Source: Sci Rep. 2016 Jan 29;7:20024. doi: 10.1038/srep20024 (PMC4731818; doi:10.1038/srep20024)
Supplement: Supplementary Information [file srep20024-s1.doc]

### Electronic supplementary material

**Kallikrein-related peptidase 8 is expressed in myocardium and induces cardiac hypertrophy**

Buqing Cao^1*^, Qing Yu^1*^, Wei Zhao^1*^, Zhiping Tang^2^, Binghai Cong^1^, Jiankui Du^1^, Jianqiang Lu^2^, Xiaoyan Zhu^1,3^, Xin Ni^1,3^

^1^Department of Physiology, Second Military Medical University, Shanghai 200433, China; School of Kinesiology, ^2^The key Laboratory of Exercise and Health Sciences of Ministry of Education, Shanghai University of Sport, Shanghai 200438, China

^*^ B.C, Q.Y. and W.Z contributed equally to this work.

^3^**Correspondence and Reprint Requests:** Dr. Xin Ni, Department of Physiology, Second Military Medical University, 800 Xiangyin Road, Shanghai 200433, China, Tel and Fax: +86-21-81870978, e-mail: [nixin@smmu.edu.cn](mailto:nixin@smmu.edu.cn); or Dr. Xiaoyan Zhu, Department of Physiology, Second Military Medical University, e-mail: xiaoyanzhu@smmu.edu.cn

### Electronic supplementary materials and methods

**Preparation of KLK8 adenovirus.** KLK8 adenovirus was generated by using the AdEasyTM adenoviral vector system (Stratagene, La Jolla, CA, USA). First, full-length rat KLK8 cDNA (GenBank accession number NM_001107509) was inserted into the pShuttle-CMV plasmid. Then pShuttle-CMV-KLK8 was linearized by PmeI digestion and recombined with pAdEasy-1 in BJ5183 cells. Correct recombinants were selected and re-transformed into Escherichia coli DH-5α, then purified and linearized with PacI digestion. This linearized recombinant vector was packaged into infectious adenoviral particles by transfecting the adenovirus packaging cell line AD-293 cells using Lipofectamine 2000 (Invitrogen). Recombinant adenoviruses were finally harvested, purified, and tittered by standard methods^51^. An empty adenoviral construct used at the same titer served as control.

**Preparation of rat neonatal cardiomyocytes culture.** Ventricle myocytes were isolated from rats that were up to 3 days old, and were isolated and cultured as described previously^55^. Briefly, ventricle tissues were minced in dissociation buffer (in mmol/L:116NaCl, 20HEPES, 0.8Na_2_HPO_4_, 5.6glucose, 5.4KCl, 0.8MgSO_4_,PH7.35) into 1mm^3^ particles. Serial digestions were performed in dissociation buffer containing 0.1% trypsin and 0.05% collagenase type II (Worthington Biochemical) at 37°C. Cell pellets were resuspended in DMEM containing 10% fetal bovine serum (FBS) and placed in culture dishes at 37°C for 1 hour to allow selective attachment of nonmyocytes (primarily cardiac fibroblasts). Cardiomyocyte-enriched fraction (>95%cardiomyocytes as determined by immunocytochemistry staining) were then seeded in 12-well culture plate (Corning, Inc. Costar Corp., Cambridge, MA) at a density of 1×10^5^ cells/cm^2^ and cultured in DMEM containing 15 mmol/L HEPES, 10% FBS, 0.1 mmol/L bromodeoxyuridine (BrdU), and antibiotics (100 U/mL penicillin and 100 mg/mL streptomycin) for 48 hours. The culture medium was then exchanged for serum-free DMEM containing the same additives with the exception of BrdU.

**RNA interference.** The small interfering RNA (siRNA) for KLK8, B_1_R, B_2_R, ERGR, PAR1 and PAR2 were designed and synthesized by GenePharma Corporation (Shanghai, China). Control siRNA was scrambled sequence without any specific target. The siRNA sequences are available in Supplemental Tab 1. Transfection of siRNA was performed by using siPORT NeoFx transfection agent (Ambion , Austin, TX）according to the instructions of the manufacturer.

**Real-Time RT-PCR.** Total RNA from heart tissue or cardiomyocytes was extracted by TRIzol reagent (Invitrogen, Grand Island, NY), and then 2µg RNA was reverse transcribed to generate cDNA by superscript reverse transcriptase (Invitrogen) according to the instructions of the manufacturer. Quantitative real-time PCR was carried out using MiniOpticon™ Real-Time PCR Detection System (BioRad, Hercules, CA). The primer sequences were designed based on cDNA sequences in GeneBank and shown in Supplemental Tab 2. The reaction solution consisted of 2.0 µl diluted cDNA, 0.2 µmol/L of each paired primer, 200 µmol/L deoxynucleotide triphosphates, 1 U Taq DNA polymerase (Qiagen, Beijing, China), and 1×PCR buffer. SYBRGreen (F Hoffmann-La Roche Ltd, Basel, Switzerland) was used as detection dye. Quantitative real-time PCR conditions were optimized according to preliminary experiment to achieve linear relationship between initial RNA concentration and PCR product. The annealing temperature was set at 58-61°C and amplification was set at 40 cycles. The temperature range to detect the melting temperature of the PCR product was set from 60°C to 95 °C. The housekeeping gene GAPDH was measured for each sample as an internal control for sample loading and normalization. To determine the relative quantitation of gene expression for both target and housekeeping genes, the comparative Ct (threshold cycle) method with arithmetic formulae (2^-△△Ct^) was used. Messenger RNA levels were normalized relative to the housekeeping gene.

**Western blot analysis.** Rat heart tissues were homogenized in cold T-Per lysis buffer, and cardiomyocytes were scraped off the plate in the presence of M-Per lysis buffer (Pierce Biotechnology). Then lysates were quickly sonified in ice bath, boiled 5 min at 100°C, and stored at -80°C until used. Equal amounts of up to 30 μg of protein samples were separated by 10% SDS-PAGE and subsequently transferred to nitrocellulose membranes. After blockage in 5% skim milk powder in 0.1% Tris-buffered saline/Tween 20 (TBST), membranes were immunostained using antibody raised against KLK1, KLK8, B_1_R, B_2_R, EGFR, PAR1 or PAR2 overnight at 4°C at a dilution range from 1:500-1000. Then, the membrane was incubated with a secondary horseradish peroxidase-conjugated antibody for 1 h at room temperature. All antibodies were purchased from Santa Cruz Biotechnology (Santa Cruz Biotechnology, Inc. Santa Cruz, CA) except for primary antibody for cleaved caspase-3 which was from Cell Signaling Technology (Beverly). Immunoreactive proteins were visualized using the enhanced chemiluminescence Western blotting detection system (Santa Cruz). The chemiluminiscent signal from the membranes was quantified by a GeneGnome HR scanner using GeneTools software (SynGene). To control sampling errors, the ratio of band intensities to the glyceraldehyde-3-phosphate dehydrogenase (GAPDH) was obtained to quantify the relative protein level.

# References

**(Note: reference numbers correspond to reference list in main article)**

1. Nicklin S.A. & Baker A.H. Simple methods for preparing recombinant adenoviruses for high-efficiency transduction of vascular cells. *Methods Mol Med.* **30,** 271-283 (1999).
2. Jian X. *et al.* MiR-204 regulate cardiomyocyte autophagy induced by hypoxia-reoxygenation through LC3-II. *Int J Cardiol.* **148**, 110-112 (2011).

Supplemental Tab1. Sequences of siRNAs used.

| Description | Sequence (5’ to 3’ ) | Accession No | |
| --- | --- | --- | --- |
| control siRNA | Forward:UUCUCCGAACGUGUCACGUTT  Reverse: ACGUGACACGUUCGGAGAATT |  | |
| KLK-8 siRNA | Forward: GGAAGGUCAGGAGUGUAAATT | NM_001107509 | |
|  | Reverse: UUUACACUCCUGACCUUCCTT |  |  |
| B_1_R siRNA | Forward: CUGCUAUCAUCUUCU UCAATT  Reverse:UUGAAGAAGAUGAUA GCAGTT | NM_030851 | |
| B_2_R siRNA | Forward:GUGGUGAACACUAUGAUAUTT  Reverse:AUAUCAUAGUGUUCACCACTT | NM_173100 | |
| EGFR siRNA | Forward:GGCAUAGGCAUUGGU GAAUTT  Reverse:AUUCACCAAUGCCUAUGCCTT | NM_031507 | |
| PAR1 siRNA | Forward: GCAGGGCAGUCUACUUAAATT  Reverse: UUUAAGUAGACUGCCCUGCTT | NM_012950 | |
| PAR2 siRNA | Forward: GUGCAUUAUUUCCUCAUCATT  Reverse: UGAUGAGGAAAUAAUGCACTT | NM_053897 | |

Supplemental Tab2. List of primers used for the amplification of various genes in rat

| Description | Sequence (5’ to 3’ ) | Product size (bp) | Accession No |
| --- | --- | --- | --- |
| GAPDH | Forward:AACGACCCCTTCATTGACCTC  Reverse: CCTTGACTGTGCGTTGAACT | 84 | NM_017008 |
| KLK-1 | Forward: AGCACCTGCCTTGCCTCA  Reverse: TTGCCTCCTTCCAACTCT | 170 | NM_001005382 |
| KLK-8 | Forward: CAGGTGGCTCGGTCTATC  Reverse: CACAGTTGAGGGTGTTTGG | 226 | NM_001107509 |
| ANP | Forward: GGGCTTCTTCCTCTTCCT  Reverse: TGAGACGGGTTGACTTCC | 246 | NM_012612 |
| BNP | Forward: TGGGCAGAAGATAGACCG  Reverse: GCAAGTTTGTGCTGGAAG | 235 | NM_031545 |
| Myh7 | Forward:GAGCCTCCAGAGTTTGCTGAAGGA  Reverse: TTGGCACGGACTGCGTCATC | 60 | NM_017240 |
| CMV-F/KLK8-R | CMV-F: CATCGCTATTACCATGGTGATG  KLK8-R: AGGCTGTCTGCCAAGGTTG | 419 |  |
| KLK8-F/IRES-R | KLK8-F: GTATGGTCTGTGCTGGCAG  IRES-R: GAAGCTTCCAGAGGAACTGC | 456 |  |


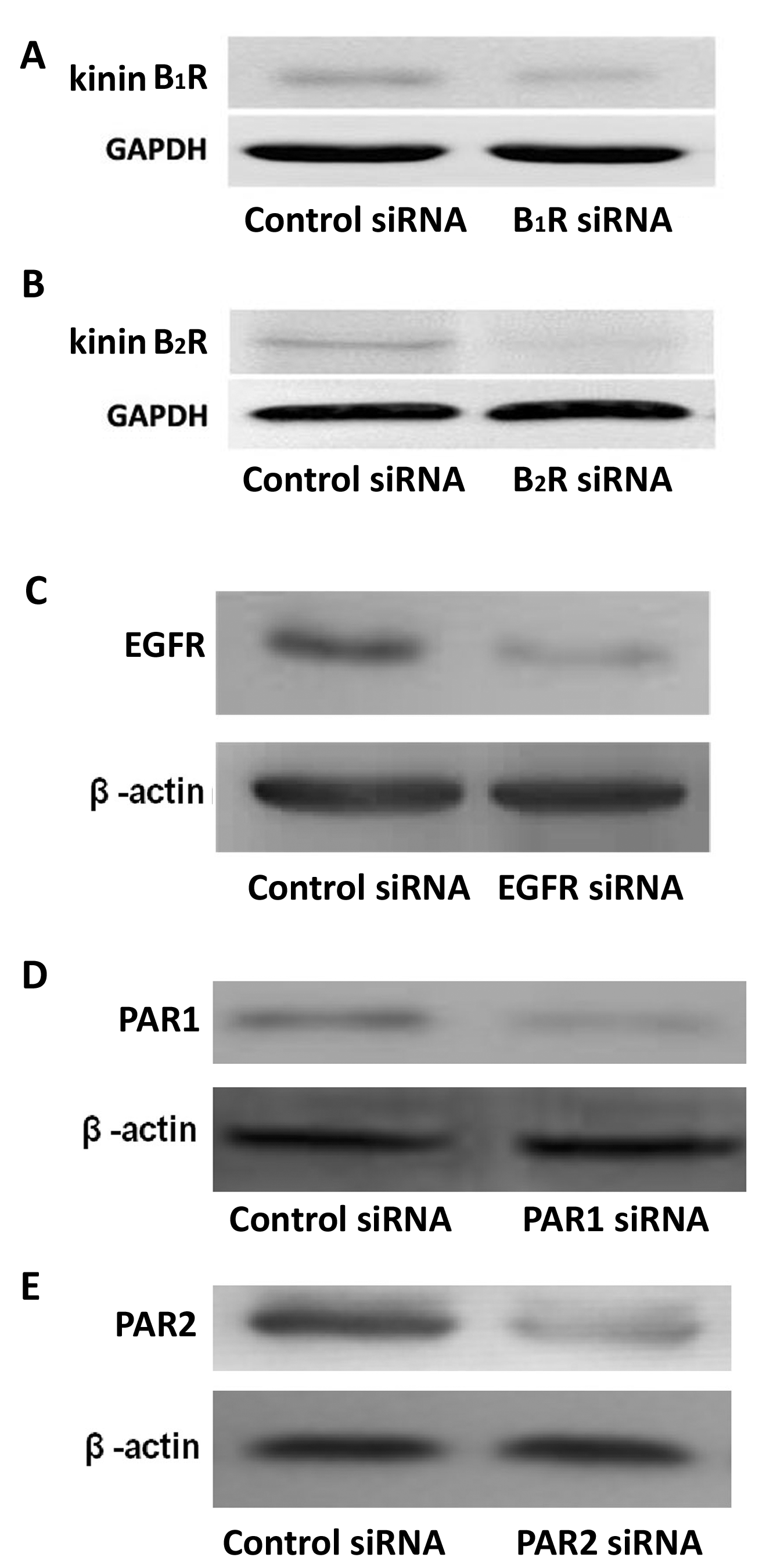


**Supplementary Figure 1:** Primary cultured neonatal rat cardiomyocytes were transfected with control siRNA or Kinin B_1_R siRNA (A), Kinin B_2_R siRNA (B), EGFR siRNA (C), PAR1 siRNA (D) and PAR2 siRNA (E). Twenty four hours later, cells were collected to determine Kinin B_1_R (A), Kinin B_2_R (B), EGFR (C), PAR1 (D) and PAR2 (E) protein levels.


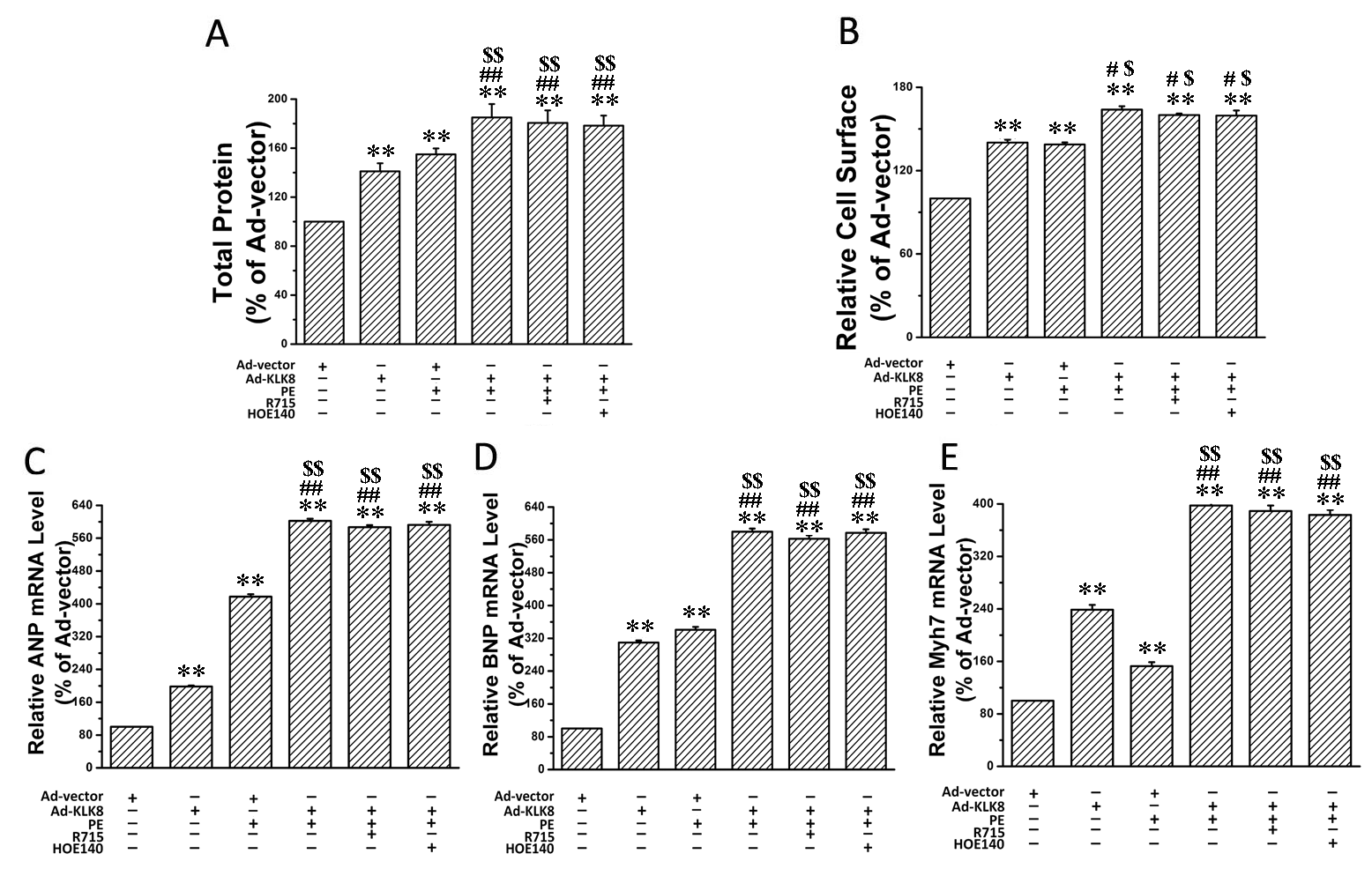


**Supplementary Figure 2: Neither kinin B_1_R antagonist, nor kinin B_2_R antagonist reverses the additive effect of Ad-KLK8 and PE on cardiomyocytic hypertrophy.** Primary cultured neonatal cardiomyocytes were infected with KLK8 adenovirus, 24 h later kinin B_1_R antagonist R715 or kinin B_2_R antagonist HOE140 was added into the culture media. After incubation for 24 h, cells were treated with PE for another 48 h. A, total protein content was determined by BCA assay; B, Cell surface area was quantified using the Image J software program; C-E, transcripts of cardiac hypertrophy markers including ANP (C), BNP (D) and Myh7 (E) were determined by quantitative real-time RT-PCR. Values are presented as mean ± SEM (n=3). ** P<0.01 vs cells treated with Ad-vector; #P< 0.05, ## P< 0.01vs cells treated with Ad-KLK8; $ P<0.05, $$ P<0.01 vs cells treated with Ad-vector + PE.

**
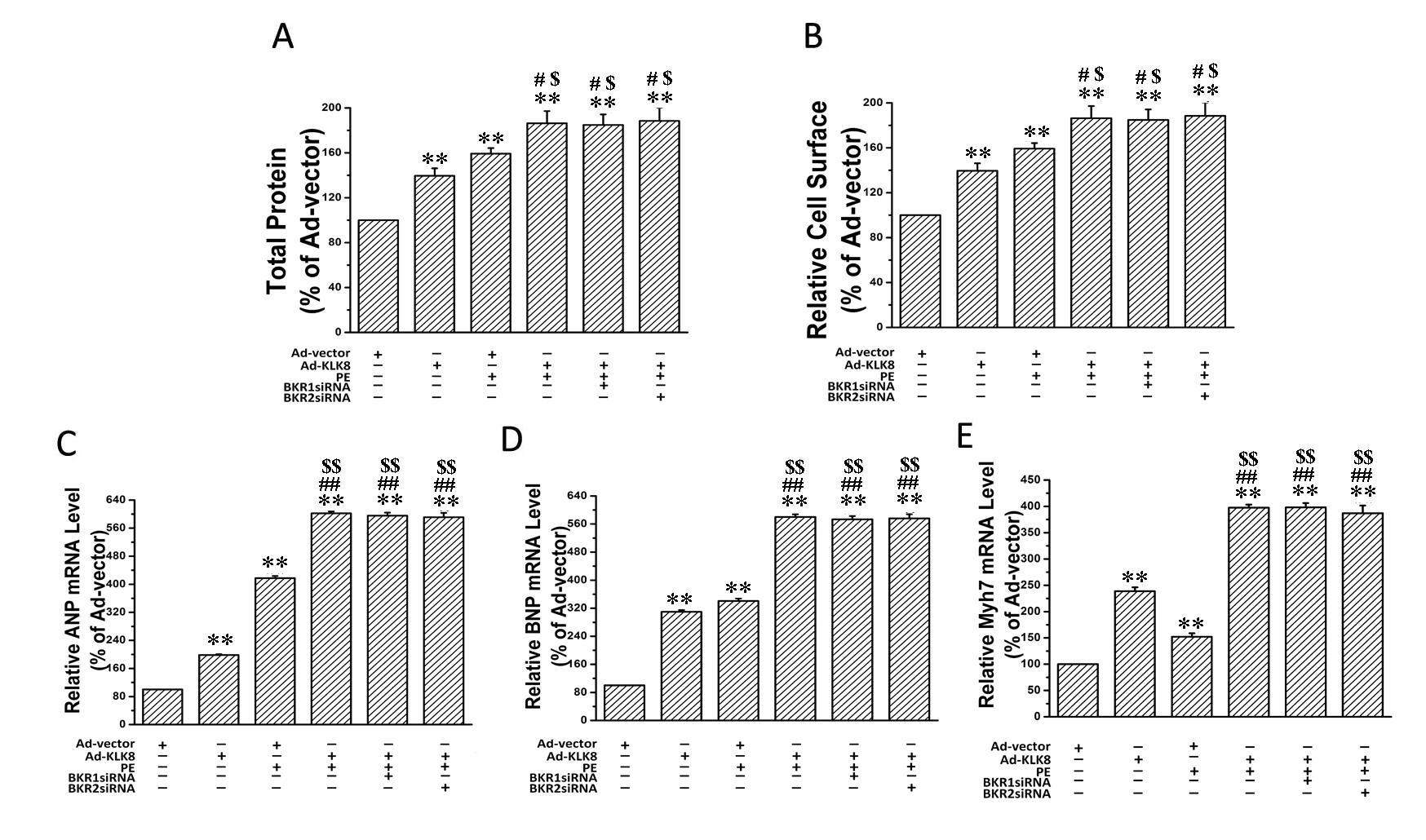
**

**Supplementary Figure 3: Neither kinin B_1_R siRNA, nor kinin B_2_R siRNA reverses the additive effect of Ad-KLK8 and PE on cardiomyocytic hypertrophy.** Primary cultured neonatal cardiomyocytes were infected with KLK8 adenovirus, 24 h later kinin B_1_R or B_2_R siRNA was transfected into the cardiomyocytes. After incubation for 24 h, cells were treated with PE for another 48 h. A, total protein content was determined by BCA assay; B, Cell surface area was quantified using the Image J software program; C-E, transcripts of cardiac hypertrophy markers including ANP (C), BNP (D) and Myh7 (E) were determined by quantitative real-time RT-PCR. Values are presented as mean ± SEM (n=3). ** P<0.01 vs cells treated with Ad-vector; #P< 0.05, ## P< 0.01vs cells treated with Ad-KLK8; $ P<0.05, $$ P<0.01 vs cells treated with Ad-vector + PE.


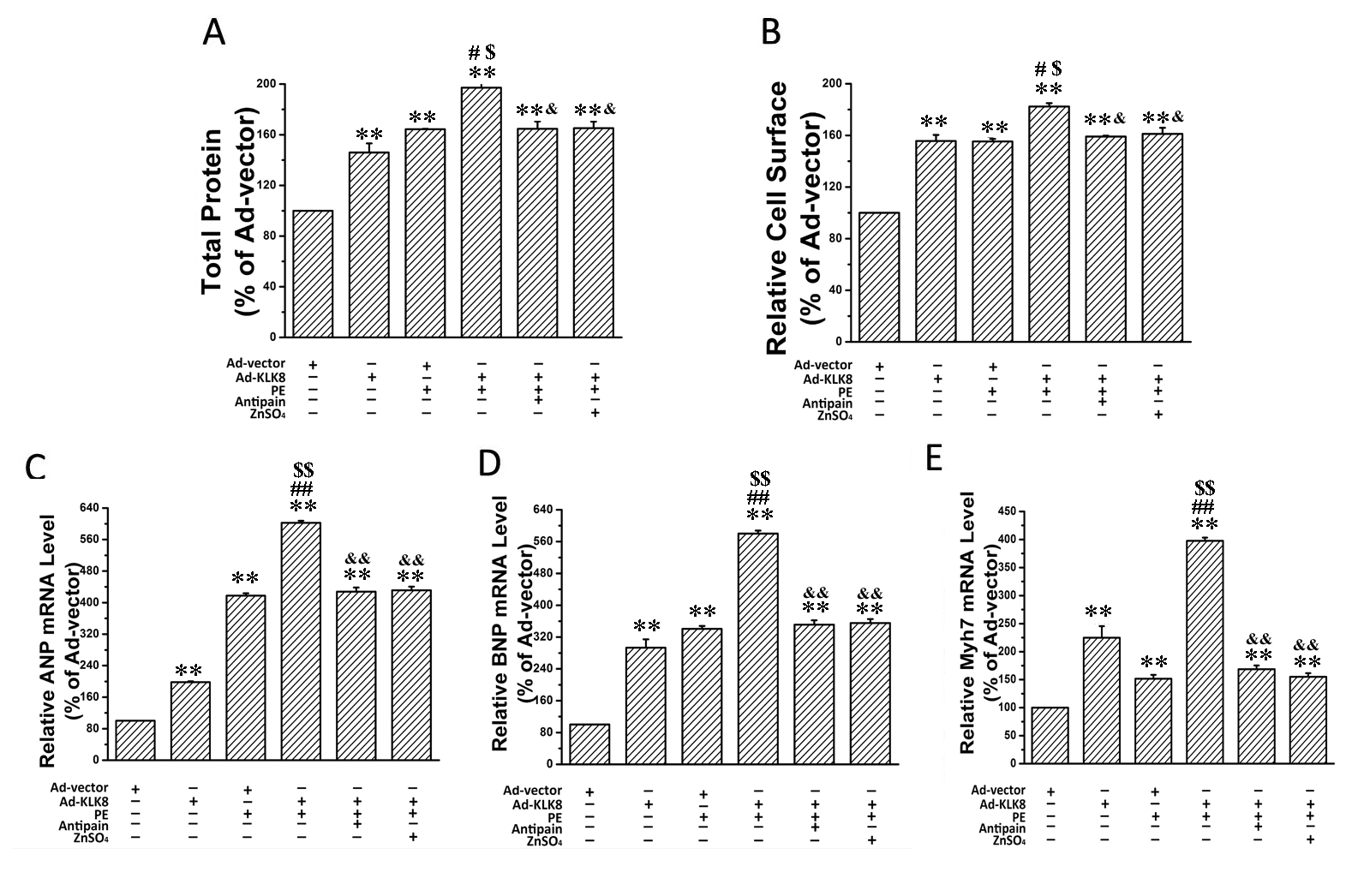
**Supplementary Figure 4: Serine protease inhibitor abolishes the additive effect of Ad-KLK8 and PE on cardiomyocytic hypertrophy.** Primary cultured neonatal cardiomyocytes were infected with KLK8 adenovirus, 24 h later serine protease inhibitor antipain or ZnSO4 was added into the culture media. After incubation for 24 h, cells were treated with PE for another 48 h. A, total protein content was determined by BCA assay; B, Cell surface area was quantified using the Image J software program; C-E, transcripts of cardiac hypertrophy markers including ANP (C), BNP (D) and Myh7 (E) were determined by quantitative real-time RT-PCR. Values are presented as mean ± SEM (n=3). ** P<0.01 vs cells treated with Ad-vector; #P< 0.05, ## P< 0.01vs cells treated with Ad-KLK8; $ P<0.05, $$ P<0.01 vs cells treated with Ad-vector and PE; & P<0.05, && p<0.01 vs cells treated with Ad-KLK8 and PE.
